# Endoscopie digestive haute à Louga (Sénégal): profil des patients et difficultés rencontrées

**DOI:** 10.11604/pamj.2017.27.211.9586

**Published:** 2017-07-20

**Authors:** Georges Antoine Bazolo Ba Ngouala, Loubna Bourgi, Joao Arm Indo Da Veiga, Arona Sakho

**Affiliations:** 1 Service de Pédiatrie, Centre Hospitalier Régional de Louga, Sénégal; 2 Service de Médecine Centre Hospitalier Régional de Louga, Sénégal; 3 Département de Médecine Hôpital Saint Jean de Dieu, Thiès, Sénégal; 4 Clinique Magou, Louga, Sénégal

**Keywords:** Endoscopie digestive haute, épi gastralgies, hernie hiatale, Upper gastrointestinal (UGI) endoscopy, epigastralgias, hiatal hernia

## Abstract

**Introduction:**

Les objectifs de cette étude étaient de déterminer le profil épidémiologique, clinique et endoscopique des patients adressés pour endoscopie digestive haute à la clinique Magou de Louga et d'analyser les difficultés rencontrées.

**Méthodes:**

il s'agit d'une étude rétrospective et descriptive qui analyse les comptes rendus de 248 gastroscopies réalisées entre le 1^er^ janvier et le 31 decembre 2014.

**Résultats:**

L'âge moyen était de 39,9 ans, le sex ratio était de 2,3 en faveur des femmes. La majorité des patients (56,5%) provenait d'une zone rurale. Les patients étaient surtout des femmes au foyer (55,2%). Les principaux prescripteurs étaient les médecins (77,8%). L'épi gastralgie était la principale indication. La hernie hiatale domine avec 33,1% suivi d'examen normaux 22,5% et le reflux gastrooesophagien isolé 12,5% des cas. Seulement 2 résultats anatomopathologiques sur 13 ont été reçus. Aucun contrôle endoscopique demandé n'a été fait.

**Conclusion:**

Peu d'endoscopie digestive haute sont réalisées à Louga. Le profil type du patient est une femme au foyer jeune vivant en zone rurale se plaignant d'épi gastralgies dont l'examen endoscopique à défaut d'être normal retrouve une hernie hiatale ou reflux gastrooesophagien. Les difficultés sont liées aux résultats d'analyses anatomopathologiques non reçus et à l'absence de contrôle endoscopique après traitement.

## Abstract (en)

**Introduction:**

This study aimed to determine the epidemiological, clinical and endoscopic profile of patients referred for upper gastrointestinal (UGI) endoscopy to the Clinique Magou in Louga and to analyze the difficulties encountered.

**Methods:**

We conducted a retrospective and descriptive study by analysing the reports of 248 gastrointestinal endoscopies performed between 1^st^ January and 31^st^ December 2014.

**Results:**

The average age was 39.9 years, with a sex ratio of 2.3 in favor of females. The majority of patients (56.5%) came from a rural area. Patients were mostly housewives (55.2%). The majority of prescribers were physicians (77.8%). Epigastralgia was the main indication. Hiatal hernia dominated (33.1%), followed by normal examinations (22.5%) and isolated gastroesophageal reflux disease (12.5%). Only 2 results out of 13 biopsies performed were reported. No requested endoscopic control was performed.

**Conclusion:**

Few upper gastrointestinal (UGI) endoscopies are performed in Louga. The typical patients’ profile is constituted by young housewives living in rural areas, complaining of gastralgies. Their endoscopic examination, instead of being normal, show hiatal hernia or gastroesophageal reflux disease. The difficulties are related to non-received anatomopathological results and to the absence of endoscopic control after treatment.

## Introduction

L'endoscopie digestive haute ou gastroscopie a été développé comme moyen de diagnostique par inspection directe du tube digestif et pour faire des biopsies. Ce rôle a été ensuite étendu à l'opacification des voies biliaires et pancréatiques. Du point de vu thérapeutique, les corps étrangers pouvaient être extraits, les rétrécissements dilatés, les polypes enlevés, la sclérothérapie et le thermo coagulation pouvaient être réalisées en toute sécurité [1]. Les objectifs de ce travail étaient de déterminer le profil épidémiologique, clinique et endoscopique des patients adressés pour gastroscopie à la clinique Magou de Louga et d'analyser les difficultés rencontrées.

## Méthodes

L'étude s'est déroulée à la clinique Magou de Louga seule structure régionale privée disposant d'une unité d'endoscopie. La région de Louga dispose d'un seul hôpital publique de niveau 2, des centres de santé, de nombreux postes de santé dirigés par des infirmiers ou des sages femmes et quelques cliniques privées. Il s'agit d'une étude rétrospective et descriptive des comptes rendus de gastroscopies faite entre le 1^st^ janvier 2014 et le 31 decembre 2014. l'examen endoscopique était pratiqué grâce a deux gastroscopes à fibres optiques Olympus GIF-E avec une anesthésie locale à la xylocaine gel visqueuse à 2%. La désinfection des endoscopes après utilisation se fait manuellement selon les procédures préconisées par la Société Française d'Endoscopie Digestive. L'âge, le sexe, la profession, l'origine des patients, les prescripteurs, les indications et les résultats de la gastroscopie ont été étudié. L'analyse des données a été faite par un logiciel EPIINFO 3.5.4.

## Résultats

Deux cent quarante huit (248) gastroscopies ont été réalisées durant la période d'étude dont 175 concernaient les femmes et 73 les hommes soit un sexe ratio de 2,3. L'âge moyen était de 39,9ans avec des extrêmes entre 2ans et 84ans. La majorité des patients (56,5%) habitait en zone rurale. La plupart des patients (55,2%) était des femmes au foyer. Les principaux prescripteurs de gastroscopie étaient les médecins (77,8%) suivi par les infirmiers (20,6%) et les sages femmes (1,6%). Les demandes d'examen étaient motivées par les signes cliniques suivants: épi gastralgies dans 62,9%, des cas, suivi de la dysphagie 17,7%, de l'hématémèse 6,9%, de la dyspepsie 6,0% des cas (Tableau 1). La pathologie œsophagienne est largement dominante avec 65,8% des cas suivie de la pathologie gastrique (6,4%) et duodénale (5,3%). Le reste était constitué d'examens normaux. Ainsi la hernie hiatale domine avec 33,1% des cas, suivi des résultats normaux (22,5%), du reflux gastro œsophagien isolé (12,5%) de la béance cardiale (6%) et de tumeurs de l'œsophage (4,4%) (Tableau 2). Les analyses anatomopathologiques demandées ne sont pas toujours faites. 13 biopsies ont été réalisées pour tumeur. Deux (2) résultats seulement rendus. Le reste n'a pas pu être acheminé au service d'anatomopathologie par les parents. De même aucun contrôle endoscopique demandé en fin de traitement n'a été fait.

**Tableau 1:** Les principales indications

Indications	nombre n=248)	pourcentage (%)
EPI gastralgies	156	62,9
Dysphagie	44	17,7
Hématémèse	17	6,9
Dyspepsie-pyrosis	15	6,0
Vomissements	10	4,0
Corps Etranger intraoesophagien	2	0,8
Ingestion de caustique	1	0,4
Hépatopathie	1	0,4
Hoquet	1	0,4
Anémie	1	0,4
Total	248	100

**Tableau 2:** Les principales affections retrouvees

RESULTATS	PATHOLOGIE	NOMBRE	POURCENTAGE
	Candidose /retro virose	2	0,8
	Hernie Hiatale	82	33,1
	Reflux gastro œsophagien	31	12,5
	œsophagite	2	0,8
	Syndrome Plummer Vinson	5	2,0
OESOPHAGE	corps étranger	2	0,8
	Sténose caustique	1	0,4
	tumeur	11	4,4
	Béance du cardia	15	6,0
	sténose du cardia	4	1,6
	varices œsophagiennes	8	3,2
	sous total	163	65,7
	Gastrite	7	2,8
	Anastomose gastroduodénal	1	0,4
ESTOMAC	Sténose du pylore	6	2,4
	tumeur	2	0,8
	sous total	16	6,5
	Duodénite	6	2,4
DUODENUM	Ulcère	7	2,8
	sous total	13	5,3
NORMAL		56	22,5
TOTAL		248	100

## Discussion

L'âge moyen des patients de notre étude était de 39,9 ans. Cette tendance jeune des malades a été retrouvée aussi dans d'autres études [2,3]. Ceci s'explique par la structure de la population qui est jeune dans notre région comme dans la plupart des pays en voie de développement. Le sex- ratio est en faveur des femmes. Ceci est retrouvée dans d'autres études [2,3] due a la fréquence élevée de troubles psychosomatiques et fonctionnels intestinaux chez les femmes. La majorité des prescripteurs étaient des médecins (77,8%) alors que dans une étude faite à Brazzaville [4] seule des médecins ou des internes en formation de spécialité médicales étaient prescripteurs. Ceci est dû à une insuffisance de ressources humaines médicales dans la région. L'indication la plus fréquente était l'épi gastralgie en rapport avec le mode d'alimentation très épicé dans la région. Ceci a été retrouvée dans des travaux similaires [2,3,5]. Par contre d'autres études ont trouvé la dyspepsie et l'hémorragie digestive haute [6-9] comme première cause d'indication de gastroscopie. Comme dans notre étude, des résultats normaux ont été retrouvé aussi dans plusieurs travaux [2,3,5,10]. Nos résultats ont montré la prédominance de la hernie hiatale avec une faiblesse de la pathologie ulcéreuse, inflammatoire et des varices 'sophagiennes alors que dans d'autres travaux la pathologie ulcéreuse et inflammatoire domine [4,6,11,12]. Cela peut être lié à la prescription intempestive des inhibiteurs de la pompe à protons par les paramédicaux sans examen endoscopique préalable. Des difficultés pratiques ont été rencontrées: difficultés diagnostiques: toutes les biopsies réalisées n'ont pas été analysées en raison du cout de l'examen qui est pris en charge par les parents; Difficultés liés au suivi: Les contrôles endoscopiques demandés après traitement ne sont pas fait une fois que le malade va bien par manque de moyens financiers et l'absence de sécurité sociale.

## Conclusion

La gastroscopie qui apporte une plus value dans les explorations du haut appareil digestif est peu réalisée à Louga. Le profil type des patients était une femme au foyer jeune, présentant des épis gastralgies dont l'examen retrouvait dans la plupart des cas une hernie hiatale, un examen normal ou un reflux gastroœsophagien. Il est important que des efforts soient faits pour sensibiliser les prescripteurs et les patients sur l'intérêt de cet examen pour le diagnostic des pathologies digestives hautes. Les difficultés énoncées ont trouvé des solutions car les examens anatomopathologiques peuvent désormais être réalisés sur place à un cout abordable et la création d'une consultation de suivi en gastroentérologie.

### Etat des connaissances actuelles sur le sujet

- C'est une méthode d'investigation qui permet de faire des biopsies, préciser le diagnostic macroscopique, faire des traitements comme la résection des polypes, détruire des tumeurs, extraire des calculs;
- Actuellement il ya un développement de la vidéo capsule qui peut après avoir été avalé fournir des images provenant de tout son trajet de la bouche à l'anus.

### Contribution de notre étude à la connaissance

- C'est la première étude en endoscopie au Sénégal hors de la capitale;
- La pathologie inflammatoire ulcéreuse n'est plus aussi importante que lors des décennies précédentes.

## Conflits d’intérêts

Les auteurs ne déclarent aucun conflit d'intérêt.
